# Gas chromatography-mass spectrometry analysis of effects of dietary fish oil on total fatty acid composition in mouse skin

**DOI:** 10.1038/srep42641

**Published:** 2017-02-14

**Authors:** Peiru Wang, Min Sun, Jianwei Ren, Zora Djuric, Gary J. Fisher, Xiuli Wang, Yong Li

**Affiliations:** 1 Department of Dermatology, University of Michigan, Ann Arbor, MI, USA; 2 Department of photomedicine, Shanghai Dermatology Hospital, China; 3 Department of Family Medicine, University of Michigan, Ann Arbor, MI, USA

## Abstract

Altering the fatty acid (FA) composition in the skin by dietary fish oil could provide therapeutic benefits. Although it has been shown that fish oil supplementation enhances EPA (eicosapentaenoic acid) and DHA (docosahexaenoic acid) abundance in the skin, comprehensive skin FA profiling is needed. We established a gas chromatography-mass spectrometry method, which allows precise quantification of FA profile using small (<24 mm^2^ for mice and <12 mm^2^ for humans) skin specimens that can be readily obtained from live mice and humans. We determined mouse skin FA composition after 2, 4 and 8 weeks of consuming a control diet or a diet supplemented with fish oil. Fish oil markedly enhanced EPA and DHA in mouse skin within 2 weeks, and this increase plateaued after 4 weeks. The FA composition in mouse skin was different from that of serum, indicating that skin has homeostatic control of FA metabolism. Mice fed the control diet designed to simulate Western human diet displayed similar skin FA composition as that of humans. The present study presents a validated method for FA quantification that is needed to investigate the mechanisms of actions of dietary treatments in both mouse and human skin.

Fatty acid (FA) composition in the skin is complex, dynamic and can be markedly affected by diet^1^,^2^,^3^,^4^. The latter is particularly applicable to essential fatty acids (EFAs)^1^,^5^, which comprise n-3 and n-6 polyunsaturated FAs that have to be obtained from diet. The n-6 FAs include linoleic acid (LA, 18:2 n-6), dihomo-linoleic acid (DGLA, 20:3 n-6) and arachidonic acid (AA, 20:4 n-6). LA can be enzymatically converted to DGLA and subsequently AA via elongation and desaturation. The n-3 FAs include alpha-linolenic acid (ALA, 18:3 n-3), eicosapentaenoic acid (EPA, 20:4 n-6), docosapentaenoic acid (DPA, 20:5 n-3) and docosahexaenoic acid (DHA, 22:6 n-3). ALA is a precursor of EPA and EPA can be further metabolized to DPA and DHA. In contrast to EFAs, saturated and monounsaturated FAs can be *de novo* synthesized by FA synthases and acquired from diet.

FA composition directly impacts properties of cell membranes, including signal transduction^6^,^7^. FAs in cell membrane phospholipids exist as esterified FA. Membrane-bound FAs give rise to a wide variety of hormone-like lipid mediators, termed as eicosanoids, upon stimulation. For instance, membrane-bound EPA and AA can be hydrolyzed by phospholipases and give rise to free EPA and free AA, both of which are catalyzed by cyclooxygenases, but generate different lipid mediators with distinct functions^8^. For instance, EPA gives rises to 3-series prostaglandins and 5-series leukotrienes, whereas AA gives rises to 2-series prostaglandins and 4-series leukotrienes^7^. Thus, the ratio of EPA versus AA is a determinant of eicosanoid production. Manipulation of EPA:AA ratios by dietary fish oil, which is rich in EPA and DHA, has been extensively studied for achieving a variety of health benefits^9^,^10^.

Dietary supplementation of fish oil is likely beneficial for prevention and treatment of several prevalent skin diseases, such as sunburn, squamous cell carcinoma and psoriasis^1^,^11^,^12^,^13^,^14^,^15^,^16^,^17^,^18^. These beneficial effects are thought to mainly result from fish oil’s impacts on FA composition and resultant changes in the skin, which justifies the investigation characterizing the effects of fish oil on skin FA composition. Among the large amount of studies that investigated fish oil effects in the context of cutaneous biology, only a handful of them quantified the incorporation of EPA and DHA into the skin^19^,^20^,^21^. While these studies have shown that fish oil intake leads to increased EPA and DHA abundance, the effects of fish oil intake on the complete FA profile in the skin remain unclear.

Precise quantification of skin FA composition is challenged by the fact that a significant proportion of cells in the skin are embedded in dense collagen fibrils in the dermis, which impedes FA extraction. The other limitation is that only a small size of skin specimen can be obtained from live human and experimental animals. These obstacles demand an effective extraction method and a sensitive quantification method to determine FA profiles in the skin.

FA quantification procedures include lipid extraction followed by transesterification of FAs into corresponding fatty acid methyl ester (FAME) via derivatization^22^. FAMEs can be separated by gas chromatography (GC) and analyzed by a flame ionization detector (FID) or an electron ionization mass spectrometry (EI-MS). Although FID is well-established and probably most commonly used for FA analysis, EI-MS has been increasingly used in recent years, especially to analyze low abundant FAs or small biological specimens since EI-MS has enhanced sensitivity and provides confirmation of analytes^10^,^22^,^23^.

Herein, we established a method of FA extraction coupled with gas chromatography-mass spectrometry (GC-MS) for quantifying skin FA profiles, which comprised eleven FAs. These eleven FAs encompassed three saturated FAs, including lauric acid (LAA, 12:0), myristic acid (MA, 14:0) and palmitic acid (PA, 16:0); and one monounsaturated FA, oleic acid (OA, 18:1n9), which are abundant in human and rodent skin. In addition, we examined four n-3 FAs and three n-6 FAs, which are directly relevant to the actions of fish oil supplementation^2^.

Stearic acid (18:0) is one of the most abundant FAs in biological samples. In our preliminary studies, we quantified stearic acid in the skin together with the eleven FAs shown in the present study. Surprisingly, we found that the abundance of stearic acid was as low as lauric acid (12:0), which was the least abundant FA examined. We also found that abundance of stearic acid was not affected by fish oil supplementation. Since the aim of our study was to investigate the influence of fish oil on the composition of major FAs, we did not include the stearic acid in the present study.

We determined time course of changes of FA compositions in the skin of mice fed a diet supplemented with or without fish oil. The diet without supplementation was designed to simulate the Western human fat intake, which contains high fat and is low in n-3 FAs. We also quantified FA composition in human skin specimens.

## Results

### FA calibration curves and precision

The FA standards were analyzed as FAMEs by GC-MS. All FA standards were well resolved. A representative chromatograph is shown in Supplemental Figure 1. The retention times and monitored ions are shown in Supplemental Table 1. Calibration curve of each standard displayed an excellent linearity with coefficients of correlation greater than 0.99 and intercepts smaller than 0.1 (Table 1). We determined the inter- and intra-day variation of each fatty acid standard at the concentration of 1.25 ug/ml, which was the lowest concentration used for making calibration curves. The inter-day relative standard deviation (RSD) ranged between 4.95% and 8.95% and the intra-day RSD ranged between 6.27% and 12.86%, suggesting that the GC-MS quantification method is precise and reproducible.

### Fatty acid composition of experimental diets

Eleven fatty acids in experimental diets were quantified using GC-MS. The FA composition (expressed as mole%) is shown in Table 2. EPA, DPA and DHA were undetectable in control diet, whereas fish oil supplemented diet contained EPA (6.9%), DPA (1.7%) and DHA (6.2%). Control and fish oil diets had similar ratio of LA versus ALA, approximately 11 to 1, which simulates the LA to ALA ratio in Western human diet.

### Fish oil supplementation markedly enhances EPA, DPA and DHA abundance at 2 weeks and these increases plateau at 4 weeks after dietary treatment

We next established a method for extracting FAs in skin specimens. Skin specimens were lysed by pulverization and a subsequent 10-minute proteinase K digestion as described in the Material and Methods section. Proteinase K digestion significantly increased FA extractability but did not affect FA composition (data not shown).

Skin FA composition was quantified at 2, 4 and 8 weeks after treatment of experimental diets (Table 3). In comparison to control diet, fish oil diet enhanced EPA, DPA and DHA abundance by 5.6 (p < 0.01), 3.9 (p < 0.01) and 5.9-fold (p < 0.01), respectively, at 2 weeks; enhanced EPA, DPA and DHA by 7.2 (p < 0.01), 7.7 (p < 0.01) and 7.7-fold (p < 0.01), respectively, at 4 weeks. These increases did not change further at 8 weeks. In addition, dietary fish oil caused changes in AA (−49%, p < 0.01), LA (−21%, p < 0.05), OA (−13%, p < 0.05) and ALA (+60%, p < 0.05) at 4 weeks and these changes were similar between 4 and 8 weeks (Table 3).

### Fish oil supplementation markedly enhances EPA, DPA and DHA abundance in the serum after dietary treatment for 8 weeks

Serum was collected from mice fed the experimental diets for 8 weeks. The results (Table 4) indicate that the trend of FA changes is similar between the skin and serum. In comparison to control serum, fish oil serum had increased EPA (30-fold, p < 0.01), DPA (4.5-fold, p < 0.01), DHA (3.8-fold, p < 0.01), ALA (1.6-fold, p < 0.01), DGLA (1.4-fold, p < 0.05) and reduced OA (−37%, p < 0.01), AA (−60%, p < 0.01).

### The changes in FA composition caused by fish oil intake differ between the skin and serum

The effects of dietary FAs on skin FA composition is secondary to its effects on FA composition in circulating blood. In order to determine the correlations in FA changes between the skin and blood, fold-changes (fish oil versus control) of FAs in the skin and serum are presented in the Table 5. The magnitudes of changes brought by fish oil between the skin and serum differed. For instance, fish oil supplementation for 8 weeks increased EPA 30-fold in serum, in contrast to 7.1-fold in the skin (Table 5).

When comparing control diet fed mice per se, there were also important differences in the FA composition between the skin and serum. The pronounced differences included: PA and OA were major FAs in the serum, compromising 29% and 28%, respectively, of total measured FAs, whereas OA was the predominant FA in the skin, and accounted for 53% of total measured FAs. In addition, AA comprised 18% of FAs in the serum, whereas it comprised 3% of FAs in skin.

### Skin FA composition in mice fed by control diet resembles FA composition in human skin

In order to compare skin FA composition between mice and humans, we quantified FA composition in skin specimens obtained from individuals who did not consume fish oil Supplements (Table 6). We found that FA composition in human skin and that of control mice was overall similar. For instance, relative OA abundance was approximate 50%; LA and PA were the second most abundant in both human and mice skin. The relative abundance of AA, EPA and DHA were also comparable between human and mice skin.

## Discussion

Skin FAs play an important role in maintaining skin homeostasis^2^. The abundance of EPA and DHA can be manipulated by dietary fish oil for potential therapeutic benefits^1^,^5^,^18^. Characterizing changes of FA composition brought by fish oil intake enhances our understanding of the actions of fish oil. However, skin FA composition has not previously been well characterized. To address his situation, we have established a GC-MS method for determination of FA composition in the skin. This method allows precise quantification of FA profiles in mouse skin specimens, i.e two punch biopsies (4 mm in diameter), which are small enough to keep mice alive without wound repair complications.

Fish oil supplementation markedly and rapidly enhanced EPA and DHA in skin at 2 weeks. Significant reduction (−60%) of AA occurred at 4 weeks. Increasing EPA and a reduction in AA presumably will alter production of bioactive eicosanoids and thus affect skin biology^3^,^5^. The increase of EPA and DHA was also associated with reduction of OA and LA, but did not alter the abundance of analyzed saturated FAs. This is likely because EPA and DHA compete with OA and LA for sn-2 positions in phospholipids, but have little effect on saturated FAs, which occupy sn-1 positions. The changes in FA composition plateaued after 4 weeks.

Our results have shown that fish oil supplementation reduces abundance of AA in both the skin and serum in comparison to the control diet. Reduced AA in the skin can be attributed to the AA reduction in the serum. AA levels in the serum are determined by diet and AA synthesis in the liver^24^,^25^. Liver-derived AA is the major source of AA in the serum. Although fish oil diet contains more AA than the control diet, the long-chain n-3 FAs, such as DHA and EPA, present in fish oil significantly suppress AA synthesis in the liver^24^,^25^, which results in reduced AA in the serum, which in turn leads to reduced AA in the skin.

In addition to extensively studied EFAs, such as EPA, DHA and AA, we have quantified DPA and DGLA. DPA, an intermediate product between EPA and DHA, has been shown to possess functions different from EPA and DHA^26^. We found that the amount of DPA was comparable to EPA and DHA in both mouse and human skin, which justifies future studies aimed at investigating the role of DPA in the skin. Dietary DGLA has been suggested to be beneficial to the skin in part by reducing AA derived eicosanoids^27^,^28^,^29^. Our results show that fish oil intake did not affect DGLA abundance, indicating that DGLA is not involved in the actions of fish oil.

We compared serum and skin FA composition in mice fed with or without fish oil for 8 weeks. We found that abundance of many FAs between skin and serum is significantly different, indicating skin controls FA accretion, which is particularly true for AA. AA comprised approximately 3% of FAs in mouse skin but 18% of FAs in mouse serum. Similarly in humans, we found that AA comprises 2.4% of FAs in human skin (Table 6) in the present study and 9% in human serum in our previous study^30^. It has been shown that AA abundance varies significantly among tissue/cells. For instance, AA comprises approximately 15%, 0.4% and 10% of measured FAs in red blood cells^31^, subcutaneous adipose tissues^32^, colonic mucosa^30^, respectively, in humans. Although caution must be exercised when comparing relative AA abundances that have been quantified in different studies because the FAs measured, subject characteristics and diets are different, these studies suggest that AA abundance appears to be particularly controlled in a tissue/cell dependent manner. The complex and specific control of AA content is consistent with its role as a precursor of an array of potent bioactive lipid mediators^7^.

The functional impacts of dietary EPA and DHA on the skin have often been investigated in mice fed diets supplemented with fish oil versus corn oil or soybean oil^33^. In the present study, we utilized a mixture of FAs that are commonly present in Western human diet as control diet^34^,^35^. We found that skin FA composition in mice fed control diet, is similar to that of human skin. This similarity supports extrapolation of results obtained from mice to humans. The method established in this study can precisely quantify FA composition using one human skin punch biopsy (4 mm in diameter). Since a 4 mm punch skin biopsy is a common clinical procedure, this method can be used for determination of skin FA composition in skin-related clinical trials. Direct quantification of skin FA composition is more accurate than extrapolating from dietary assessment.

The aim of the present study was to establish an accurate and reliable method that can quantify changes of FA profiles brought by fish oil treatment using small skin specimens. Such a method is a prerequisite for future investigations into the potential beneficial effects of fish oil supplementation on prevention and treatment of certain skin disorders. These investigations would be performed on patients and/or animal models with defective skin. Healthy animals were utilized in the present study. Fish oil did not cause histological alteration in healthy mouse skin, indicating that fish oil does not possess unwanted side effects on healthy skin. The method established using healthy skin provides a reference for pertinent studies of dermatological research.

To our knowledge, comprehensive FA profiles in human skin have not been reported previously, although FA compositions in the sebum have been studied^36^,^37^. However, FAs in the sebum and skin cells are nearly completely different with respect to synthesis, composition and function. All sebum FAs are exclusively produced by the sebocytes in the sebaceous glands^37^. Sebocytes possess enzymatic machineries that are capable of producing a unique mixture of FAs, which is largely different from composition of FAs present in a cell. Sebum is secreted to the skin’s surface to moisturize skin. In contrast, FA composition in a cell is determined by both cellular properties and diet, For instance, EFAs have to be acquired by diet. Some EFAs, such as AA, EPA and DHA, can give rise to a variety of hormone-like lipid signaling molecules, which regulate diverse biological processes^25^.

The processes of collection and extraction of FAs from the sebum and skin cells are completely different. Sebum on the skin’s surface can be readily collected using non-invasive methods, such as tape striping^37^. Lipid is the predominant constituent in the sebum, and thus can be directly extracted by an organic solvent. In contrast, FAs inside skin cells have to be acquired from skin specimens, which have to be obtained by a surgical procedure, i.e. biopsy. Only a small size of biopsy can be obtained to reduce potential adverse effects, such as wound healing problems. The size of biopsy limits the amount of FAs. Furthermore, FAs are minor components of the skin. Skin is primarily composed of protein, such as collagen, in terms of mass. Thus a process that degrades protein and maximizes FA extraction is desirable. In the present study, we established a method that can quantify FA composition in skin cells using a small skin specimen.

The differences between FAs in the sebum and skin cells described above dictate the research focuses on sebum and skin FAs are different. Our main focus was to determine the changes of AA, EPA, DPA, DHA brought by fish oil treatment. In addition, we examined how fish oil affects major FAs in skin cells. We focused on eleven major FA species because FAs extracted from a small skin specimen are not sufficient for detecting FAs with low abundance. In contrast, sebum which is much more accessible than skin biopsies can be collected in a sufficient amount to allow determination of FAs of trace amount^36^. Therefore, different FA species were chosen to be examined between our study and studies concerning sebum lipids^36^.

It has been shown that dietary fish oil can reduce triacylglycerol (DAG) in circulating blood in part by reducing the production of very low density lipoprotein (VLDL) in the liver^38^. In the skin, DAG is primarily produced by the sebocytes and is a major component of sebum^39^. The potential impact of fish oil supplementation on DAG levels in the sebum is not known and is of interest for future investigation.

FAs in cells predominantly exist as acyl chains in phospholipids. Thus, fish oil treatment-induced changes of FAs shown in this study reflect the alterations of esterified FAs bound with phospholipids. Since esterified AA, EPA and DHA in membrane phospholipids can be hydrolyzed to free FAs, which can be further converted to various lipid signaling molecules^25^, fish oil could regulate skin function by altering skin phospholipids.

## Materials and Methods

### Animals and diets

Female C57BL/6 mice were purchased from Charles River Laboratory (Wilmington, MA). Mice were housed five per cage under a 12 hour light–dark cycle and maintained at 22 ± 1 °C. Mice were randomized to receive a control or fish oil diet, which was custom compounded by Dyets Inc. (Bethlehem, PA). The compositions of diets were adapted from our previous studies^34^,^35^. Briefly, the diets were modified AIN-93 diets with lower cornstarch to accommodate a higher fat content. Both diets were isocaloric per kg and contained 34% of total calorie from fat. Both diets had the same composition (shown as percentage by weight) of casein (22%), cornstarch (25%), dyetrose (15.5%), sucrose (10%), cellulose (5%), t-Butylhydroquinone (0.0034%), mineral mix (3.9%), vitamin mix (1%), L-cysteine (0.3%), choline bitartrate (0.28%). Control diet contained Western Blend fat (17%), whereas fish oil diet contained Western Blend fat (10%) and menhaden oil (7%). The Western Blend fat was composed of the following oils: coconut (45%), olive (30%), corn (15%) and soybean (10%).

Mice were fed one of these two diets ad libitum for various times. Diet was stored at −80 °C. Approximately five grams of the respective diet were dispensed daily per animal. At the end of the study, animals were sacrificed by isoflurane inhalation and decapitation. No significant difference in body weights has been observed in animals receiving either of the diet. All animal experiments were conducted in accordance with the guidelines of the National Institutes of Health. All animal protocols were approved by the University Committee on Use and Care of Animals at the University of Michigan.

### Human skin tissue procurement

All procedures involving human subjects were approved by the University of Michigan Institutional Review Board and were carried out according to the principles of the Declaration of Helsinki. Written informed consent was obtained from each subject. Volunteers who did not consume fish oil supplements were recruited. The age and sex of five volunteers were: 54, female; 30, female; 44, male; 59, male; and 33, male; respectively. Skin samples (4 mm in diameter) were obtained from buttock/hip by punch biopsies and stored in −80 °C until processing as described previously^40^,^41^.

### Chemicals and Standards

Fatty acid standards were purchased from NuChek Prep (Elysian, MN). These included: dodecanoic (12:0, lauric acid, LAA), tetradecanoic (14:0, myristic acid, MA) hexadecanoic (16:0, palmitic acid, PA), octadeca-9-enoic (18:1 n-9, oleic Acid, OA), octadeca-9,12-dienoic acid (18:1 n-9, linoleic acid, LA), octadeca-9,12,15-trienoic (18:3 n-3, alpha-Linolenic acid, ALA), eicosa-8,11,14-trienoic (20:3 n-6, dihomo-γ-linolenic acid, DGLA), eicosa-5,8,11,14-tetraenoic (20:4 n-6, arachidonic acid, AA), eicosa-5,8,11,14,17-pentaenoic (20:5 n-3, eicosapentaenoic acid, EPA), docosa-7,10,13,16,19-pentaenoic (22:5 n-3, docosapentaenoic acid, DPA), docosa-4,7,10,13,16,19-hexaenoic (22:6 n-3, docosahexaenoic acid, DHA). The margaric acid (17:0) was used as an internal standard.

MethPrep II (methanolic m-trifluoromethylphenyltrimethylammonium hydroxide) derivatization reagent was obtained from Alltech Inc. (Deerfield, IL). All other reagents were purchased from Sigma-Aldrich (St. Louis, MO).

### Generation of calibration curves

Each FA standard was dissolved in hexane:dichloromethane (1:1) at concentrations ranging from 0.5–2 mg/ml to make stock solutions. Stock solutions then were diluted to make six mixtures of standards with concentrations ranging from 1.25–50 μg/ml. The concentration of the internal standard (17:0) was kept constant at 10 μg/ml in all samples. Standard mixtures were derivatized and analyzed by GC-MS as described below. The calibration curves were calculated by linear regression. The intra-day and inter-day relative standard deviations (RSD) were quantified using standards with concentration of 1.25 μg/ml, which was the lowest concentration used for making calibration curves. Injection volume was 1 μl.

### Fatty Acid Extraction

At various times after feeding the experimental diets, mice were anesthetized and hair was removed using an electric clipper. Skin specimens (4 mm in diameter) were obtained using a biopsy punch (Acuderm Inc, Ft.Lauderdale, FL). Two frozen skin punch specimens were homogenized using a metal pulverizer (MultiSample Bio-Pulverizer, Research Product International, Mount Prospect, IL). Pulverized samples were placed in 400 μl PBS and sonicated by Ultrasonic Cell Disruptor (Qsonica Misonix Inc., Farmingdale, NY) followed by proteinase K (200 ng/ml) incubation at 56 °C for 10 minutes to lyse skin tissue and cells. The resultant lysate was centrifuged at 12,000 rpm to remove residue hair and tissue debris that were resistant to protease K digestion. To extract fatty acids, supernatant (200 μl) were mixed with the solvent composed of dichloromethane (800 μl), methanol (400 μl) and water (200 μl)^22^. The solvent contained margaric acid (17:0) (10 μg) as an internal standard. Samples were vigorous vortexed for 1 minute followed by centrifuging for 5 minutes at 8,000 rpm using a benchtop centrifuge. The dichloromethane layer at the bottom was removed carefully to avoid taking precipitated protein in the interface. Dichloromethane, which contained extracted lipids, was dried under nitrogen flow with heating at 37 °C. Dried samples were dissolved in the solvent composed of dichloromethane (800 μl), methanol (200 μl) and water (200 μl), followed by the same FA extraction to further remove non-lipid constituents.

The same method was used for FA extraction from human skin specimens. FAs extracted from one human skin biopsy (4 mm in diameter) were sufficient for quantification by GC-MS. Mouse blood samples were obtained using cardiac puncture. Fatty acids were extracted from serum (50 μl) using the method described above.

### Preparation of FAMEs

Dried fatty acid extracts were derivatized by adding 70 μl of hexane:dichloromethane (1:1) and 30 μl Meth-Prep II. Samples were vortexed briefly and incubated at room temperature for 30 minutes to allow formation of FAMEs. FAs exist as esterified and free FAs in a cell. The transesterification procedure used by our study converted both esterified and free FAs into FAMEs. Thus, the method utilized in our study examined total FAs.

### Quantification of FAMEs by gas chromatography-mass spectrometry (GC-MS)

GC-MS method was adapted from our previous publication^22^. Briefly, GC was performed use an Agilent 6890 N GC equipped with an Agilent 7683 N autosampler and a Supelco-SP2330 column (30 m × 0.25 mm × 0.2 μm film thickness). The injector was set at 220 °C using the splitless injection mode, and 1 μl injections were made. GC was run using an optimized temperature program as follows: The GC temperature program started at 100 °C followed by a linear increase to 176 °C at 10 °C/min, a slower linear increase to 185 °C at 1.5 °C/min, followed by an increase to 225 °C at 12 °C/min. The total run time was 20 minutes. Helium was the carrier gas. The flow rate through the column was 2.5 ml/min. FAMEs separated by GC were detected by MS (Agilent 5973) using single ion monitoring mode. The ions monitored and their retention times are shown in Supplemental Table 1. Peak area ratio relative to that of the internal standard, i.e. 17:0, was referred to calibration curves to quantify each fatty acid.

### Statistical analysis

The significances of differences between groups of samples were determined by ANOVA followed by post hoc analysis using Tukey’s test. Differences were considered significant when the p-value was less than 0.05. Data are expressed as mean ± standard error of mean (SEM).

## Additional Information

**How to cite this article:** Wang, P. *et al*. Gas chromatography-mass spectrometry analysis of effects of dietary fish oil on total fatty acid composition in mouse skin. *Sci. Rep.* **7**, 42641; doi: 10.1038/srep42641 (2017).

**Publisher's note:** Springer Nature remains neutral with regard to jurisdictional claims in published maps and institutional affiliations.

## Supplementary Material

Supplementary Dataset 1

## Figures and Tables

**Table 1:** Calibration curves of fatty acid standards.

Fatty acids	Calibration curve equation	Coefficient correlation (r^2^)	Intra-day RSD%	Inter-day RSD%
LAA (12; 0)	y = 0.0549x − 0.0607	0.9969	4.55	8.87
MA (14; 0)	y = 0.0539x + 0.0340	0.9930	5.32	6.27
PA (16; 0)	y = 0.0994x − 0.0458	0.9988	7.64	11.60
OA (18; 1 n-9)	y = 0.0596x + 0.0531	0.9952	8.95	11.70
LA (18; 2 n-6)	y = 0.0319x − 0.0007	0.9948	6.52	14.73
ALA(18; 3 n-3)	y = 0.1331x − 0.0569	0.9959	7.67	12.86
DGLA (20; 3 n-6)	y = 0.2217x − 0.0268	0.9975	6.71	9.44
AA (20; 4 n-6)	y = 0.2663x + 0.0915	0.9966	5.09	9.67
EPA (20; 5 n-3)	y = 0.1431x − 0.0113	0.9961	8.22	10.71
DPA (22; 5 n-3)	y = 0.0902x + 0.0715	0.9938	8.95	11.50
DHA (22; 6 n-3)	y = 0.0771x − 0.0684	0.9933	7.20	12.00

**Table 2:** Fatty acid composition (in mole %) of experimental diets.

Diet	Control diet	Fish oil diet
LAA (12; 0)	16.2 ± 0.9	12.6 ± 0.1*
MA (14; 0)	8.5 ± 0.3	8.4 ± 0.0
PA (16; 0)	14.6 ± 0.6	15.3 ± 0.1
OA (18; 1 n-9)	45.1 ± 0.5	33.5 ± 0.4*
LA (18; 2 n-6)	15.9 ± 0.4	15.2 ± 0.5
ALA (18; 3 n-3)	1.3 ± 0.1	1.4 ± 0.2
DGLA (20; 3 n-6)	U.D.	U.D.
AA (20; 4 n-6)	U.D.	1.2 ± 0.4
EPA (20; 5 n-3)	U.D.	6.9 ± 0.4
DPA (22; 5 n-3)	U.D.	1.7 ± 0.1
DHA (22; 6 n-3)	U.D.	6.2 ± 0.9
LA/ALA	11.5	10.7

U.D.: undetectable. Data shown is mean and SEM for five different aliquots of diets. *Asterisk indicates p < 0.05 when comparing the fish oil and control diet.

**Table 3:** Skin fatty acid composition (mole %) after feeding experimental diets for 2, 4 and 8 weeks (n = 5).

Mouse Skin	2-week		4-week		8-week	
	control	fish oil	control	fish oil	control	fish oil
LAA (12; 0)	1.7 ± 0.5	1.9 ± 0.3	3.9 ± 0.2	3.9 ± 0.5	3.5 ± 0.3	2.9 ± 0.3
MA (14; 0)	3.0 ± 0.5	3.3 ± 0.3	3.5 ± 0.3	3.5 ± 0.3	3.4 ± 0.2	3.0 ± 0.4
PA (16; 0)	18.2 ± 2.1	16.5 ± 1.3	13.3 ± 1.2	13.2 ± 1.1	15.4 ± 0.9	13.2 ± 1.1
OA (18; 1 n-9)	50.5 ± 3.6	47.3 ± 2.0	51.4 ± 2.3	45.1 ± 3.4	53.1 ± 2.1	47.1 ± 2.0
LA (18; 2 n-6)	21.4 ± 2.8	19.0 ± 2.6	20.2 ± 2.0	16.5 ± 1.2	18.0 ± 1.3	16.0 ± 1.1
ALA (18; 3 n-3)	0.9 ± 0.2	1.2 ± 0.2	0.9 ± 0.2	1.4 ± 0.2	0.9 ± 1.2	1.3 ± 1.1
DGLA (20; 3 n-6)	0.4 ± 0.0	0.4 ± 0.1	0.3 ± 0.1	0.2 ± 0.0	0.4 ± 0.1	0.4 ± 0.1
AA (20; 4 n-6)	2.8 ± 0.5	2.1 ± 0.3	3.5 ± 0.6	1.7 ± 0.2	3.0 ± 0.4	1.6 ± 0.2
EPA (20; 5 n-3)	0.4 ± 0.1	2.2 ± 0.2	0.5 ± 0.1	3.8 ± 0.4	0.5 ± 0.1	3.2 ± 0.2
DPA (22; 5 n-3)	0.6 ± 0.1	2.5 ± 0.3	0.6 ± 0.1	4.5 ± 1.4	0.7 ± 0.1	4.5 ± 0.5
DHA (22; 6 n-3)	0.7 ± 0.1	4.0 ± 0.5	0.8 ± 0.2	6.9 ± 1.3	0.7 ± 0.0	6.5 ± 0.4

**Table 4:** Serum fatty acid composition (mole %) after feeding experimental diets for 8 weeks (n = 5).

Mouse Serum	Control	Fish oil
LAA (12; 0)	2.5 ± 0.1	2.6 ± 0.3
MA (14; 0)	3.4 ± 0.5	3.7 ± 0.5
PA (16; 0)	29.2 ± 0.6	30.3 ± 1.2
OA (18; 1 n-9)	28.4 ± 1.2	18.0 ± 1.1
LA (18; 2 n-6)	14.3 ± 0.4	13.2 ± 0.5
ALA (18; 3 n-3)	0.4 ± 0.0	0.6 ± 0.1
DGLA (20; 3 n-6)	0.7 ± 0.0	1.0 ± 0.1
AA (20;4 n-6)	18.0 ± 1.2	8.3 ± 0.7
EPA (20; 5 n-3)	0.3 ± 0.0	9.0 ± 0.6
DPA (22; 5 n-3)	1.0 ± 0.1	4.6 ± 0.4
DHA (22; 6 n-3)	2.7 ± 0.2	10.2 ± 0.9

**Table 5:** Fold-changes (fish oil versus control) of FAs in the skin and serum after feeding experimental diets for 8 weeks (n = 5).

	skin	serum
	Fish oil/Control	Fish oil/Control
LAA (12; 0)	0.9 ± 0.1	1.0 ± 0.1
MA (14; 0)	0.9 ± 0.1	1.1 ± 0.1
PA (16; 0)	0.9 ± 0.1	1.0 ± 0.0
OA (18; 1 n-9)	0.9 ± 0.0	0.6 ± 0.0**
LA (18; 2 n-6)	0.9 ± 0.0	0.9 ± 0.0
ALA (18; 3 n-3)	1.4 ± 0.1	1.6 ± 0.1
DGLA (20; 3 n-6)	0.9 ± 0.1	1.4 ± 0.1*
AA (20; 4 n-6)	0.5 ± 0.1	0.5 ± 0.0
EPA (20; 5 n-3)	7.1 ± 0.8	30.3 ± 1.2**
DPA (22; 5 n-3)	6.7 ± 0.6	4.5 ± 0.5**
DHA (22; 6 n-3)	8.9 ± 0.5	3.8 ± 0.2**

*p < 0.05, **p < 0.01 when comparing fold-changes between the skin and serum presented in the Table 5.

**Table 6:** Fatty acid composition (mole%) in human skin (n = 5).

	Human skin
LAA (12; 0)	1.4 ± 0.4
MA (14; 0)	2.7 ± 0.7
PA (16; 0)	22.3 ± 1.3
OA (18; 1 n-9)	50.1 ± 2.6
LA (18; 2 n-6)	18.4 ± 1.2
ALA (18; 3 n-3)	1.4 ± 0.4
DGLA (20; 3 n-6)	0.4 ± 0.1
AA (20; 4 n-6)	2.4 ± 0.7
EPA (20; 5 n-3)	0.4 ± 0.1
DPA (22; 5 n-3)	0.3 ± 0.0
DHA (22; 6 n-3)	0.8 ± 0.1
