# Supplementary material for: Gas chromatography-mass spectrometry analysis of effects of dietary fish oil on total fatty acid composition in mouse skin
Source: Sci Rep. 2017 Feb 14;7:42641. doi: 10.1038/srep42641 (PMC5307384; doi:10.1038/srep42641)
Supplement: Supplementary Dataset 1 [file srep42641-s1.doc]

**Gas chromatography-mass spectrometry analysis of effects of dietary fish oil on total fatty acid composition in mouse skin**

Peiru Wang^1,3^, Min Sun^1^, Jianwei Ren^2^, Zora Djuric^2^, Gary J Fisher^1^, Xiuli Wang^3,*^, Yong Li^1,*^

^1^Department of Dermatology, ^2^Department of Family Medicine, University of Michigan, Ann Arbor, MI, USA. ^3^Department of photomedicine, Shanghai Dermatology Hospital, China.

^*^ to whom correspondence should be addressed.

**Supplemental data**

Supplemental Table 1, GC-MS parameters for detection of FAMEs using single ion monitoring

|  |  |  |  | |  |  | |  |
| --- | --- | --- | --- | --- | --- | --- | --- | --- |
| Fatty acid | | | | Retention time  (Minute) | | | Ion (m/z) monitored | |
| LAA (12;0) | | | | 5.42 | | | 183 | |
| MA (14;0) | | | | 6.93 | | | 199 | |
| PA (16;0) | | | | 8.52 | | | 199 | |
| OA (18;1 n-9) | | | | 10.95 | | | 298 | |
| LA (18;2 n-6) | | | | 11.91 | | | 294 | |
| ALA (18;3 n-3) | | | | 13.18 | | | 292 | |
| DGLA (20;3 n-6) | | | | 15.38 | | | 222 | |
| AA (20;4 n-6) | | | | 15.79 | | | 203 | |
| EPA (20;5 n-3) | | | | 16.65 | | | 201 | |
| DPA (22;5 n-3) | | | | 18.48 | | | 208 | |
| DHA (22;6 n-3) | | | | 18.86 | | | 199 | |
| Internal standard (17:0) | | | | 9.41 | | | 241 | |


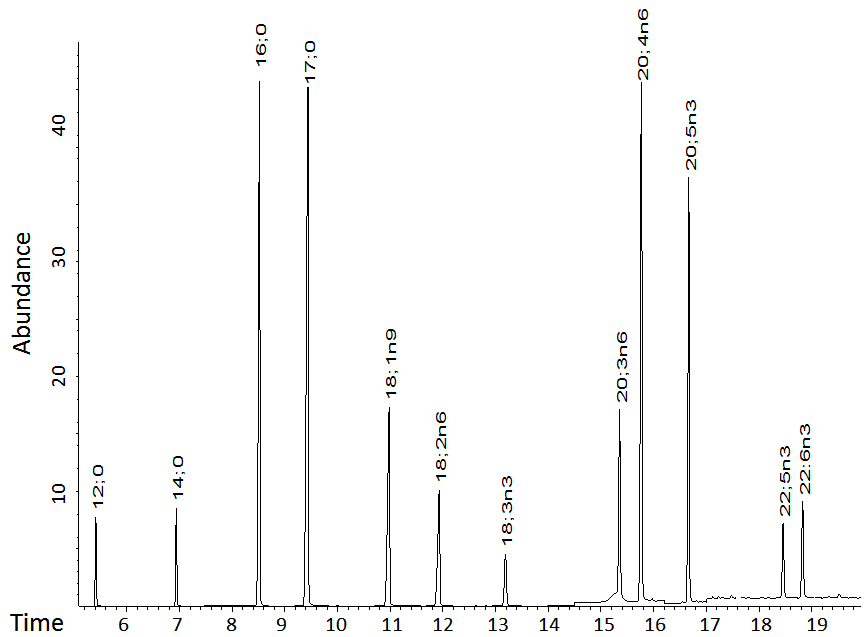


Supplemental Figure 1, representative GC-MS chromatograph of FA standards
